# SmilODB: a multi-omics database for the medicinal plant danshen (*Salvia miltiorrhiza*, Lamiaceae)

**DOI:** 10.3389/fpls.2025.1586268

**Published:** 2025-05-20

**Authors:** Yong-Hui Liu, Wen-Qiong Zeng, Si-Fan Tao, Yi-Nuo Du, Fei-Hong Yu, Zhe-Chen Qi, Dong-Feng Yang

**Affiliations:** Zhejiang Province Key Laboratory of Plant Secondary Metabolism and Regulation, College of Life Sciences and Medicine, Zhejiang Sci-Tech University, Hangzhou, China

**Keywords:** *Salvia miltiorrhiza*, multi-omics database, gene expression, transcriptomics, 3D structure, deep learning

## Abstract

**Introduction:**

*Salvia miltiorrhiza* Bunge (Danshen) is a traditional medicinal plant widely used in the treatment of cardiovascular and inflammatory diseases. Although various omics resources have been published, there remains a lack of an integrated platform to unify genomic, transcriptomic, proteomic, and metabolomic data.

**Methods:**

To address this gap, we constructed the *S. miltiorrhiza* Multi-omics Database (SmilODB, http://www.isage.top:56789/), which systematically integrates publicly available genome assemblies, transcriptome datasets, metabolic pathway annotations, and protein structural predictions. Protein structures were predicted using the RoseTTAFold algorithm, and all data were visualized using interactive heat maps, line charts, and histograms.

**Results:**

SmilODB includes: (i) two genome assemblies of *S. miltiorrhiza*, (ii) 48 tissue-specific transcriptome datasets from root, leaf, and other vegetative tissues, (iii) annotated biosynthetic pathways for bioactive compounds such as tanshinones and salvianolic acids, and (iv) 2,967 high-confidence protein models. The database also integrates bioinformatics tools such as genome browsers, BLAST, and gene heatmap generators.

**Discussion:**

SmilODB provides an accessible and comprehensive platform to explore multi-omics data related to *S. miltiorrhiza*. It serves as a valuable resource for both basic and applied research, facilitating advances in the understanding of this medicinal plant’s molecular mechanisms and therapeutic potential.

## 1 Introduction

*S. miltiorrhiza* is a perennial herb of the genus Salvia in the Lamiaceae family. It is a traditional Chinese medicinal herb with a clinical history spanning over 2,000 years, first recorded in the classical Chinese medical text Shen Nong’s Herbal Classic (Wang et al., 2020a). According to the Pharmacopoeia of the People’s Republic of China (Chinese Pharmacopoeia Commission, 2020), *S. miltiorrhiza* is indicated for invigorating blood circulation, removing blood stasis, relieving pain, calming the mind, and cooling blood to reduce abscesses. Modern studies have shown that the active components in *S. miltiorrhiza* can be classified into two main components: lipophilic tanshinones, including tanshinone I, tanshinone IIA, tanshinone IIB, dihydrotanshinone I, cryptotanshinone, and hydrophilic phenolic acids, such as salvianolic acid (DSU), caffeic acid (CA), rosmarinic acid (RA), salvianolic acid A (Sal A), and salvianolic acid B (Sal B) (Jiang et al., 2019; Shi et al., 2019). Due to its remarkable medicinal value, *S. miltiorrhiza* is widely used around the world for the treatment of numerous diseases, including coronary heart disease, cerebrovascular diseases, Alzheimer’s disease, Parkinson’s disease, kidney deficiency, liver cirrhosis, cancer, and osteoporosis (Chong et al., 2019; Guo et al., 2020; Lee et al., 2020; Wang et al., 2020b; Sun et al., 2021; Xu et al., 2022; Huang et al., 2024).

Due to the significant medicinal value of *S. miltiorrhiza*, extensive research has been conducted on the genes, proteins, and metabolites associated with its medicinal components, especially those involved in the biosynthesis of tanshinones and phenolic acids (Li et al., 2015; Ma et al., 2015; Zhang et al., 2015). Transcriptomic studies have identified key genes regulating these biosynthetic pathways, such as *SmCPS1*, *SmKSL1* (Jia et al., 2018; Pei et al., 2018; Li et al., 2019; Liu et al., 2020; Yu et al., 2020), and various transcription factors (e.g., *WRKY*, *bHLH*) (Bai et al., 2018; Xing et al., 2018a, 2018b; Yu et al., 2018; Zhang et al., 2020a, 2021). While these transcriptomic discoveries identify candidate regulators, elucidating the enzymatic functions and ligand-binding mechanisms of their protein products remains critical for understanding the biosynthesis and pharmacological activity of tanshinones. Additionally, research has focused on the related regulatory mechanisms and networks (Yang et al., 2018; Zhang et al., 2020b). Moreover, only 21 *S. miltiorrhiza* protein structures have been experimentally resolved in the Protein Data Bank (PDB, https://www.rcsb.org/), leaving most of its predicted proteome structurally unannotated. This gap critically limits rational engineering of metabolic pathways for enhanced medicinal compound production. To address this, deep learning-based protein modeling now offers transformative solutions. With the advancement of deep learning technologies, protein structure and function prediction has seen significant progress. For instance, AlphaFold2 (Bryant et al., 2022) has significantly improved the accuracy of protein structure prediction, DynamicBind (Lu et al., 2024) aids in predicting the formation of protein-ligand complexes, and ESM3 (Hayes et al., 2025), as a multimodal generative language model, can infer protein sequences, structures, and functions.

Despite substantial advances in *S. miltiorrhiza* multi-omics data—encompassing genomic, transcriptomic, metabolomic, and AI-predicted protein structural models—a critical bottleneck persists: the absence of a unified platform for systematic integration and multimodal analysis. This fragmentation not only impedes research efficiency but also constrains comprehensive exploration of the plant’s biological characteristics and pharmacological mechanisms. Consequently, developing a centralized data platform to enable researchers to cohesively access and interrogate these heterogeneous datasets has emerged as an urgent priority.

Currently, existing plant multi-omics databases, such as the Plant Metabolic Network (PMN) (Hawkins et al., 2021), the Arabidopsis Information Resource (TAIR) (Lamesch et al., 2012), and Integrated Medicinal Plantomics (IMP) (Chen et al., 2024), provide valuable resources for plant science research, However, they primarily focused on model plants or a limited number of medicinal plants and do not provide detailed multi-omics information specifically for *S. miltiorrhiza*. Similarly, specialized Traditional Chinese Medicine (TCM) databases, such as the Traditional Chinese Medicine Integrated Database (TCMID) (Huang et al., 2018) and Traditional Chinese Medicine Plant Genome (TCMPG) (Meng et al., 2022), include extensive of compound and target information for various TCM herbs. However, omics-level data for *S. miltiorrhiza* in these databases remains limited. The SmGDB developed by Zhou et al (Zhou et al., 2022), integrates genomic data for *S. miltiorrhiza* but still faces certain limitations. Primarily, SmGDB focuses on genomic and transcriptomic data integration while lacking detailed analysis of metabolic pathways and dynamic changes of key secondary metabolites, such as tanshinones. Additionally, while it provides gene expression data, SmGDB does not include advanced tools for protein function prediction, which is critical for understanding the biological roles of gene products.

These limitations underscore the necessity of developing a dedicated multi-omics database for *S. miltiorrhiza*. This study aims to establish such a database by integrating genomics, transcriptomics, metabolomics data, along with protein structure predictions using deep learning models. Through this database, researchers will gain rapid access to relevant genetic information, transcriptomic profiles, metabolic pathway data, and three-dimensional protein structures, thereby advancing pharmacological mechanism research and clinical applications of *S. miltiorrhiza*.

## 2 Materials and methods

### 2.1 Genomic data processing

To provide a high-quality genomic foundation for SmilODB, we improved the existing *S. miltiorrhiza* genome assembly previously published by Xu et al. (2016), which originally consisted of 21,045 scaffolds with a Contig N50 of only 12.38 Kb and a Scaffold N50 of 51 Kb. The high level of fragmentation limited the accurate resolution of key biosynthetic gene clusters, such as those encoding cytochrome P450 (CYP450) enzymes involved in tanshinone biosynthesis. To address these limitations, we performed a *de novo* reassembly of the *S. miltiorrhiza* genome by incorporating high-throughput chromosome conformation capture (Hi-C) data to enhance genome scaffolding and chromosomal anchoring. Scaffolding was performed using the 3D-DNA pipeline (Dudchenko et al., 2017), which generated chromosome-level assemblies by integrating Hi-C interaction maps. The improved assembly resulted in a total genome size of 514.41 Mb and a Scaffold N50 of 58.54 Mb.

From this newly assembled genome, we extracted 27,729 annotated gene sequences. Additionally, we incorporated the 595 Mb genome assembly of the *S. miltiorrhiza* DSS3 line published by Song et al. (2020), from which we extracted 29,236 annotated gene sequences. We processed the genome assembly results in FASTA format as follows: First, individual gene sequences were extracted using the getfasta command from bedtools (Quinlan and Hall, 2010) based on the genome annotation file. Then, CDS and protein sequences for each gene were obtained using gffread (Pertea and Pertea, 2020). Next, we calculated the sequence length, N content, GC percentage, and gene count for each chromosome. Since the IDs in the extracted gene FASTA files were based on genomic coordinates, a custom Python script was used to match annotation-derived position information to assign correct gene IDs. All processed genomic data, including gene ID, chromosomal location, coordinates, sequence, and length, were organized in tabular format and integrated into SmilODB for public access and query.

### 2.2 Transcriptome data processing

This study utilized a total of 48 transcriptomic datasets, which include samples from various tissues of *S. miltiorrhiza*: whole tissues (4 samples), roots (22 samples), stems (2 samples), flowers (15 samples), and leaves (5 samples). Thirteen of these datasets were provided by our research team (unpublished), while the remaining 35 datasets were obtained from the NCBI, SRA database (including PRJNA437195, PRJNA771193, and PRJNA757189). Among these, the PRJNA757189 project consists of single-end sequencing data, while the rest are paired-end sequencing data.

After obtaining the raw reads, we performed quality control using Trimmomatic v0.32 (Bolger et al., 2014) to remove low-quality sequences and adapter contamination from the FASTQ files. This filtering method helps reduce errors and noise in the sequencing data, improving the reliability of subsequent analyses. After quality control, the data were assessed using FASTQC to generate a quality report. We also calculated the Q20 and Q30 scores using a shell script to evaluate the sequencing accuracy. To address potential batch effects arising from the integration of transcriptomic datasets from different sources, we performed quality control and batch correction. Principal Component Analysis (PCA) was initially used to visualize sample clustering and detect potential batch-driven variation. Subsequently, we applied the ComBat function from the sva package in R (Leek et al., 2012) to adjust for known batch effects (i.e., project origin and sequencing type), while preserving biologically meaningful variation. This correction was conducted prior to downstream analyses such as differential expression and clustering. The use of ComBat, an empirical Bayes method, is widely recommended for removing batch-associated variation in high-throughput genomic data while retaining biological signals (Johnson et al., 2007).

For transcriptome analysis, we employed a reference-based assembly approach using the improved *S. miltiorrhiza* reference genome described in Section 2.1 (514.41 Mb; Scaffold N50: 58.54 Mb) as the reference. First, we constructed the genome index using HISAT v2 2.1.0 (Kim et al., 2015) and aligned individual FASTQ sample files to the reference genome using the -dta parameter, which ensures that the results are compatible with StringTie v2.2.3 (Pertea et al., 2016) for transcript assembly. After aligning the reads to the reference genome, SAM files containing alignment information for each sample were obtained. The SAM files were then converted into BAM files and sorted using SAMtools-1.9 (Li et al., 2009). The sorted BAM files were used as input for the StringTie tool to perform transcript assembly and merging, with the results generated in a specified GTF file. Subsequently, StringTie was used to quantify the abundance of each gene and transcript, producing expression files for each sample.

### 2.3 Gene expression and metabolic pathway annotation

Gene expression may vary across different plant tissue types and developmental stages. Therefore, in this study, we performed a statistical analysis of gene expression in various tissues and organs of *S. miltiorrhiza* to reveal the expression patterns and functional relationships of key genes. Gene expression levels were evaluated using TPM, which normalizes for gene length and sequencing depth, eliminating their effects on gene abundance calculations. To minimize differences between projects and samples, the research team used Python scripts to extract TPM values from different samples and performed a ${\text{log}}_{2}\left(\text{TPM}+1\right)$ transformation to make the data more concentrated and suitable for subsequent analysis. We then conducted pathway annotation, focusing on the biosynthetic pathways of important compounds in *S. miltiorrhiza*, including tanshinones, salvianolic acids, flavonoids, and plastoquinones. The expression levels of key enzyme genes in these pathways were visualized in heatmaps to compare expression patterns across different samples. Through these analyses, this study reveals the sources and synthesis mechanisms of various medicinal components in *S. miltiorrhiza*, providing valuable information for the study of its pharmacological effects.

### 2.4 Protein tertiary structure prediction

To ensure biological relevance and technical reliability, we selected 2,967 high-confidence protein sequences for structure prediction based on the following stringent criteria: (1) experimentally validated expression in *S. miltiorrhiza* under elicitor treatments (Ag⁺, methyl jasmonate [MJ], or fosmidomycin [FOS]); (2) known or predicted functional association with the tanshinone biosynthetic pathway; and (3) significant differential expression based on transcriptomic data. These proteins were prioritized as candidates most likely to be involved in specialized metabolite biosynthesis.

For tertiary structure modeling, we employed RoseTTAFold (Abramson et al., 2024), which provides an optimal balance between accuracy and computational efficiency—particularly for large-scale predictions in non-model plant species. Compared to AlphaFold2 and ESMFold, which offer high accuracy but require substantial computational resources, RoseTTAFold enabled broader coverage of the *S. miltiorrhiz*a proteome within the constraints of available infrastructure. Benchmarking studies have shown that RoseTTAFold performs comparably well for many plant proteins with curated inputs (Stephan et al., 2021). The prediction process followed RoseTTAFold’s hybrid pipeline: Initially, a homologous sequence search is conducted to generate Multiple Sequence Alignments (MSA), which are then used for template searching. Subsequently, the obtained template information is preprocessed, and feature information is updated in the 2D track. Finally, the 3D structure of the protein is initialized and optimized in the 3D track. In the prediction of protein folding, RoseTTAFold offers two methods: (1) pyRosetta Method (Chaudhury et al., 2010): This method uses the inferred distance map as folding constraints, folding 15 conformations and selecting the top five best results. (2) SE(3)-Transformer Method (Fuchs et al., 2020): This method iteratively optimizes the backbone coordinates, generating a 3D structure that contains only the backbone, until convergence criteria are met, known as the end-to-end model. After comparing the results on the CASP14 test set, the pyRosetta method was ultimately selected for predicting and optimizing protein folding. In addition, we used the ProtBert pre-trained model to encode protein sequences and extract features., followed by the use of Python scripts to generate molecular graphs of the protein structure. These graphs’ feature matrices and adjacency matrices are then input into the Graph Attention Network (GAT) (Veličković et al., 2017) for feature fusion and updating. Finally, the output is passed through fully connected layers to generate probabilities for GO term functional annotations. Since protein folding occurs in three-dimensional space, methods relying solely on sequence feature extraction have inherent limitations. However, by employing GAT, we can more effectively capture spatial features of protein folding, enabling more accurate functional predictions. The predicted protein structures were ultimately visualized through integration of the Mol* Viewer for interactive 3D structural representation and analysis.”

### 2.5 Development of database and website

The multi-omics database for *S. miltiorrhiza* constructed in this study integrates various advanced technologies aimed at achieving efficient data storage, management, and visualization. The database backend utilizes MySQL 8.0 relational database (https://www.mysql.com) for data storage, built with the Django framework. The system employs the Model-View-Controller (MVC) architecture pattern to separate business logic, data, and interface, and integrates Django Rest Framework (DRF) for developing RESTful APIs, thereby improving the efficiency and maintainability of API development. The frontend is based on Vue 2.0 framework and Element UI component library, providing responsive design and a good user experience, supporting dynamic data display and interface layout optimization. To implement sequence alignment functionality, the database integrates the BLAST and SequenceServer (Priyam et al., 2019), allowing users to submit homologous sequence alignment requests for genomes and proteins via a web interface and obtain alignment results quickly. For genome data visualization, the JBrowse (Buels et al., 2016) genome browser is integrated, enabling users to browse, search, and analyze genomic and annotation data. Moreover, the MolStar (Sehnal et al., 2021) tool is used for visualizing 3D protein structures, providing enhanced insights into the protein foldings. Through multiple optimizations of the database architecture, this system effectively enhances the efficiency and stability of data processing, providing users with efficient analysis features and a user-friendly interface, thereby promoting the in-depth advancement of bioinformatics research related to *S. miltiorrhiza*.

## 3 Results

**Figure 1:**
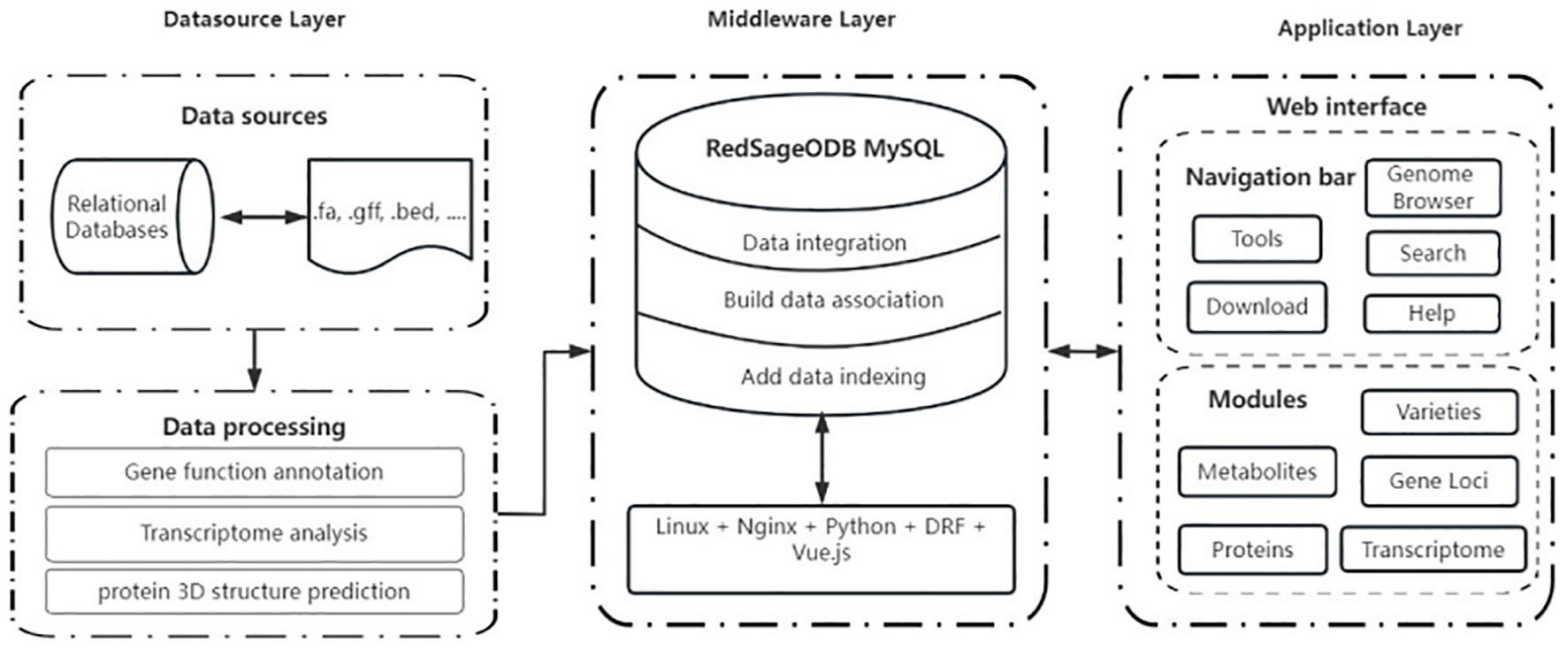
Architecture of SmilODB, including data source layer, middleware layer, and application layer.

**Figure 2:**
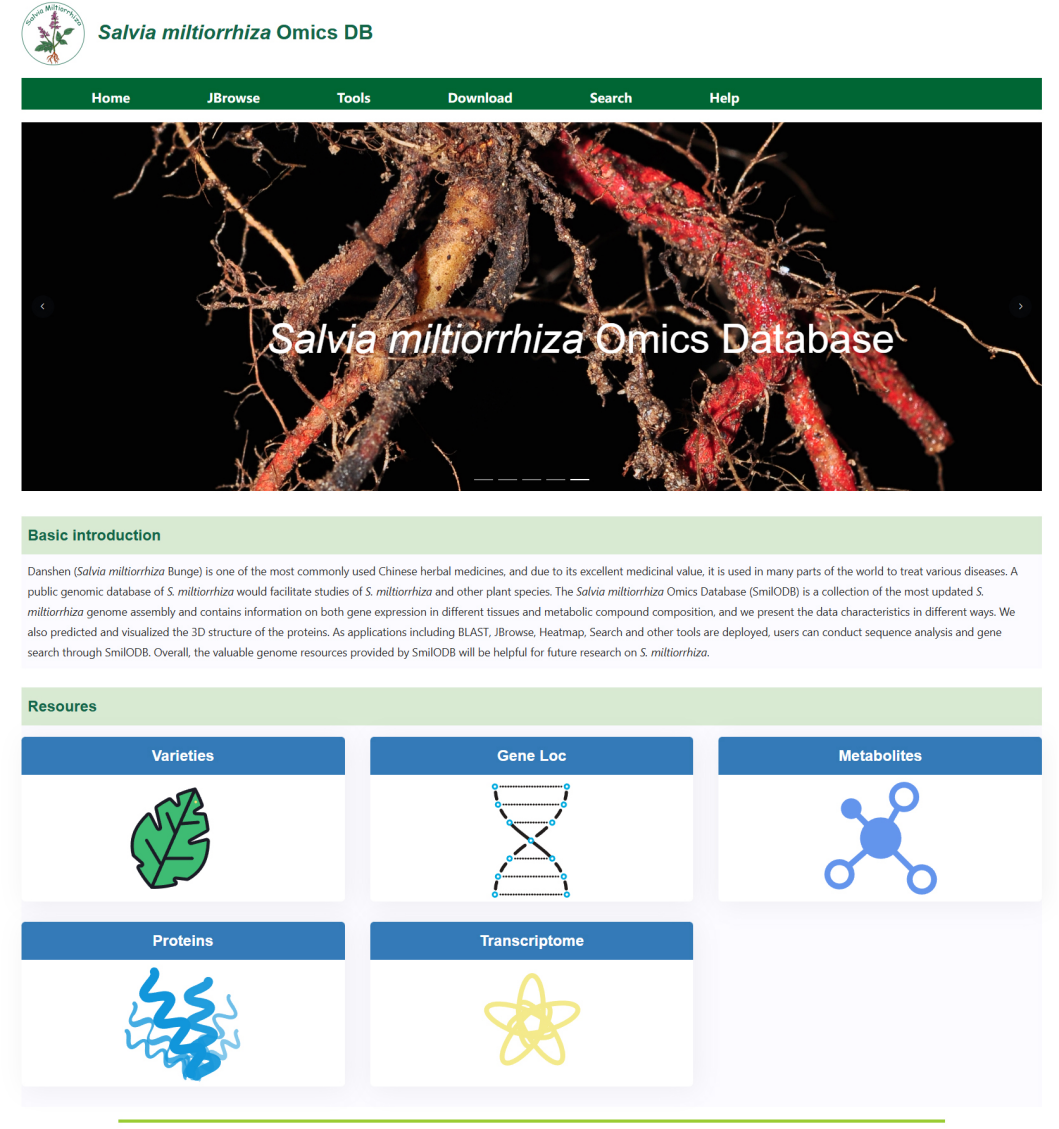
The homepage of the database provides access points to various features.

The structure of SmilODB is shown in **Figure 1**. The database component consists of several parts, including gene function annotation, transcriptome analysis, and protein 3D structure prediction. The front-end and back-end interaction is built using Django and Vue, and the user interface is divided into two sections. As shown in **Figure 2**, the navigation section includes all the tools available on the website, such as the JBrowse genome browser, download module, search module, BLAST [35] tool, heatmap tool, and the compound analysis tool in the toolbox. The functional section comprises five omics modules, summarizing the analytical data for *S. miltiorrhiza* across various omics groups: varieties, gene loci, metabolites, proteins, and transcriptomes.

### 3.1 Varieties module

**Figure 3:**
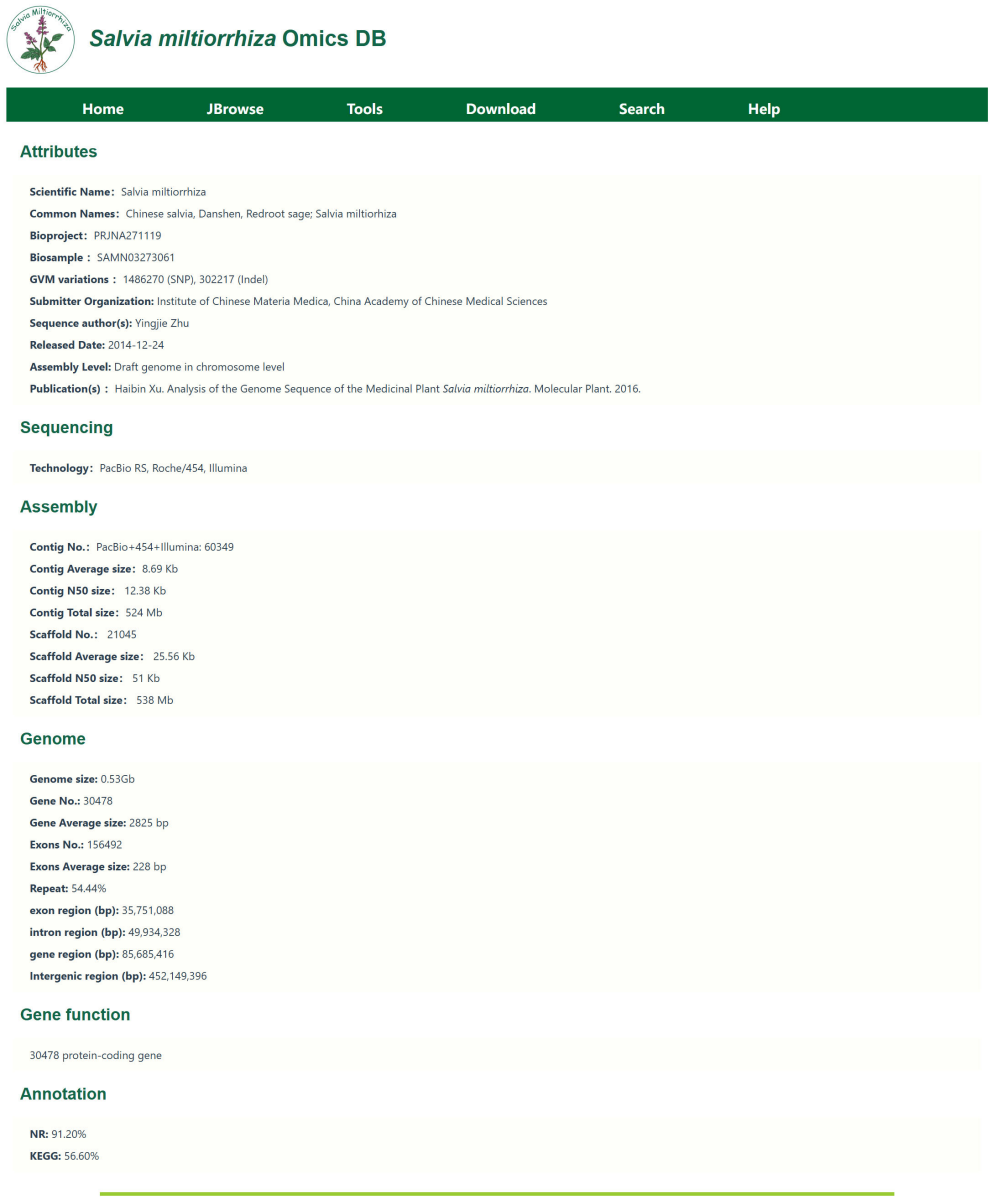
Gene sequencing information. Including information such as chromosome length, N content, GC content, and gene number.

The Varieties Module primarily introduces genome sequencing information. The genome sequencing information is shown in **Figure 3**.This page presents basic information such as the genome size and repeat rate for the two *S. miltiorrhiza* species currently included in the database. Clicking on the lines name allows access to detailed information about the genome assembly, including sequencing methods, sequencing technologies, assembly data, protein-coding genes, and functional annotation summaries. For example, for the DSS3 line, the genome was sequenced by Song et al. (2020), with a genome coverage of 0.97, 1,487 contigs, 982 scaffolds, and GC content of 37.8%. This module provides existing genomic resources for *S. miltiorrhiza* and assists researchers in gaining a comprehensive understanding of the sequencing information, as well as facilitating comparative studies across different lines.

### 3.2 Gene locus module

**Figure 4:**
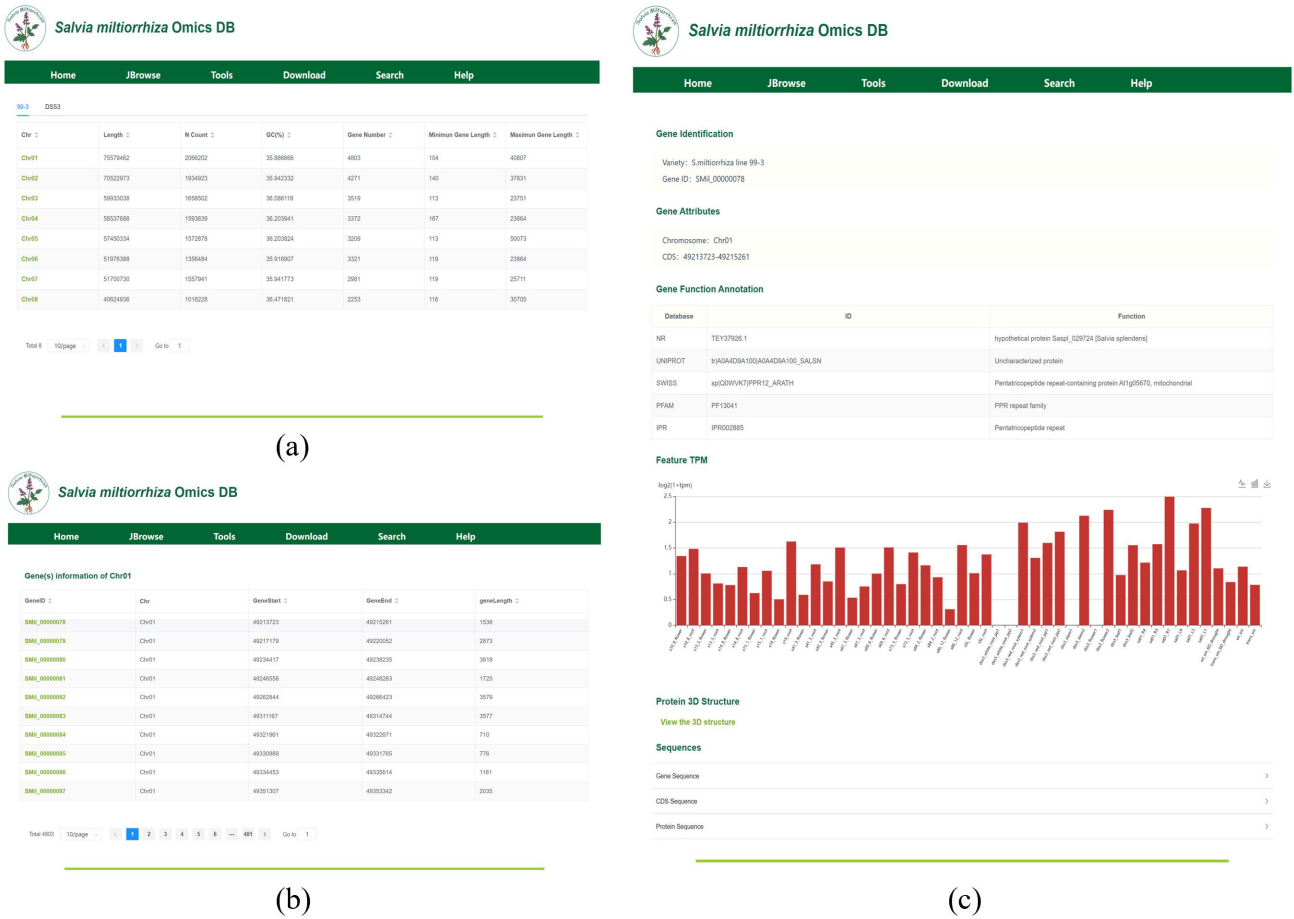
**(a)** The interface displays chromosomal metrics (length, GC content, gene density) for cultivar ‘99-3’. **(b)** Gene Information on Chromosome (Scaffold), display of 10 genes (of 4,803 total) on chromosome 01, showing genomic coordinates (GeneStart, GeneEnd, geneLength) and unique identifiers (GeneID). **(c)** Key Gene Information: Includes key information such as gene variants, attributes, functional annotations, expression levels, and 3D structures.

The Gene Locus Module displays gene information on each chromosome or scaffold of *S. miltiorrhiza* and provides detailed annotations for individual genes. Upon clicking the module, users will be directed to a page displaying the chromosome or scaffold information for each genome, as shown in **Figure 4a**. In the tab bar, users can choose to view different genomes. This page also includes statistics on key information such as chromosome length, N content, GC content, and the number of genes. By clicking on a chromosome or scaffold name, users can access the gene information page for that specific chromosome, as shown in **Figure 4b**. The gene annotation file provides detailed information for all genes on the chromosome, including their corresponding chromosome name, gene length, and start and stop positions. For instance, Chr01 harbors 4,803 genes, including SMil_00000078 (Chr01:49,213,723-49,215,261), a 1,539-bp gene encoding a diterpene synthase. By clicking on a gene ID, users will be taken to the gene detail page, as shown in **Figure 4c**. This page presents detailed information such as the gene’s species, gene attributes, functional annotations, transcript expression levels, and 3D structure. Additionally, users can view the corresponding FASTA format sequences for the gene’s CDS, protein, and nucleotide sequences.

### 3.3 Transcriptome module

**Figure 5:**
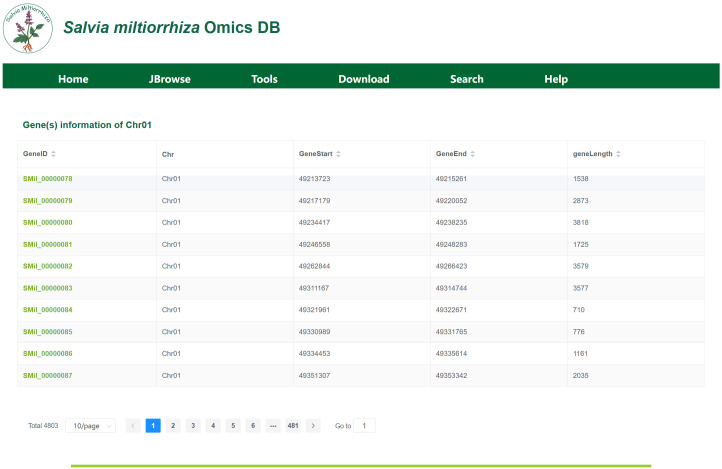
Gene expression levels are presented in three forms: table, gene heatmap, and line graph.

To present gene expression levels more intuitively, we developed the transcriptome information module using transcript expression data from 48 samples. In the “Transcriptome” module (as shown in **Figure 5**), we provide two search methods: by gene ID or by gene region. After searching by gene ID, the system displays the transcript expression levels in three formats: a table, a gene heatmap, and a line chart.

The table presents the specific TPM (Transcripts Per Kilobase of exon model per Million mapped reads) values, the gene heatmap visually shows the gene expression patterns across samples and the differences between them, and the line chart allows users to observe the overall expression of genes across different samples, making it easy to understand the gene expression levels. For example, the gene SMil_00000975 has higher expression in flower tissues, while the gene SMil_00000082 shows more significant expression in root tissues.

### 3.4 Metabolites module

**Figure 6:**
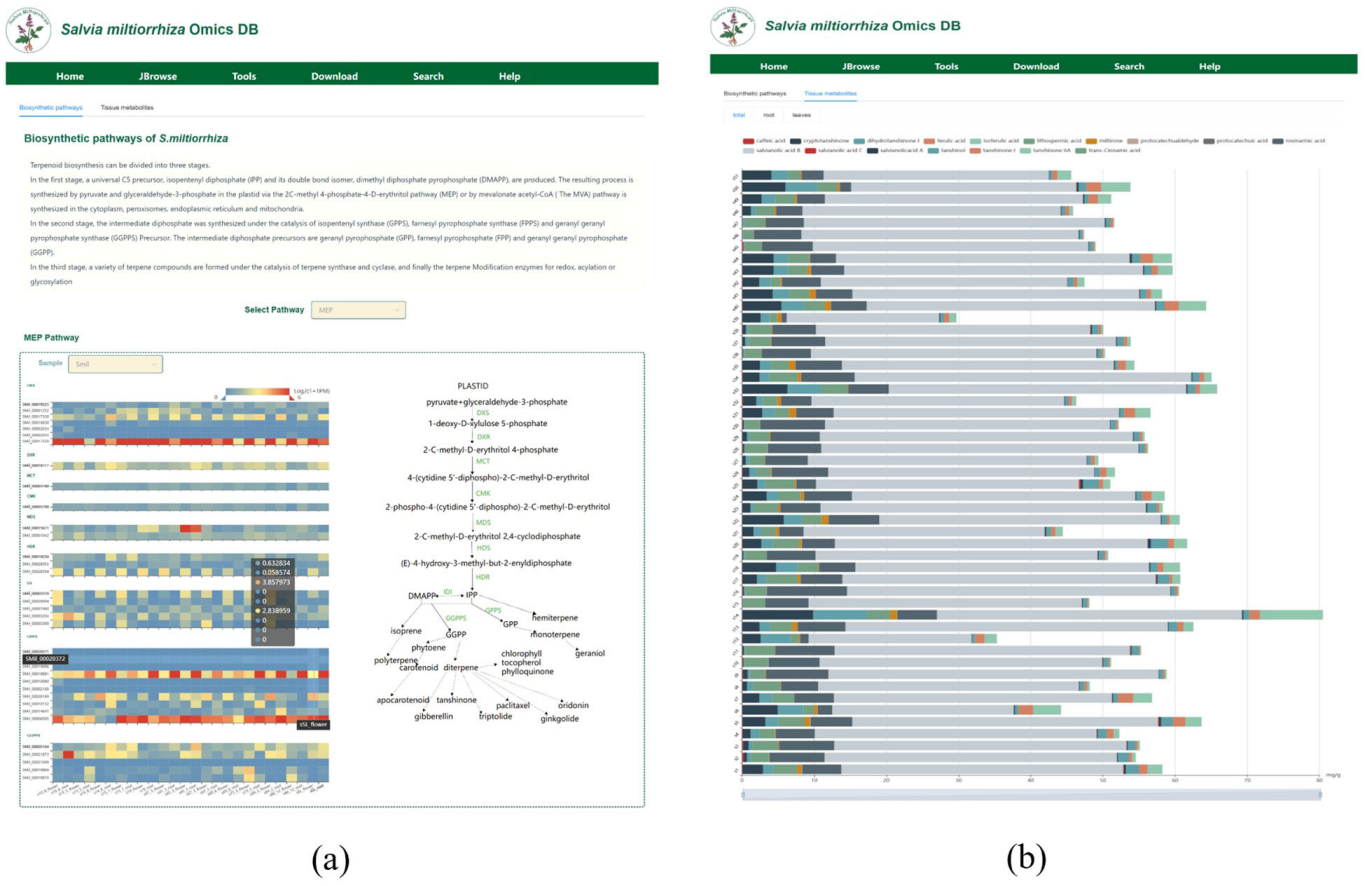
Metabolite module interface **(a)** Metabolic pathway annotation: Displays metabolic pathways and gene expression across different sample groups. **(b)** Compound content: Dynamic charts visualize the expression levels of key compounds.

The Metabolites module is divided into two subsections: ‘Biosynthetic Pathways’ and ‘Tissue Metabolites’. In the “Biosynthetic pathways” section (as shown in **Figure 6a**), we first introduce the three stages of the upstream MEP(Methylerythritol 4-Phosphate) and MVA(Mevalonate) pathways. Then, we integrate the annotations for six metabolic pathways from the three projects and present the gene expression data for different pathways and sample groups in dynamic charts. In the “Select Pathway” dropdown menu, users can select the name of the metabolic pathway, including the MEP pathway, MVA pathway, downstream pathways of salvianolic acid, flavonoid pathway, plastoquinone and ubiquinone pathways. The corresponding metabolic pathway and the gene expression heatmap of key enzymes for that pathway will be displayed below. By default, the expression data for 26 samples are shown. Additionally, users can also select different samples in the “Sample” section to observe their expression patterns. The database is designed to be expandable through data uploads, with corresponding results and visualizations updated in real-time.

In the “Metabolites” module, under the “Tissue metabolites” section (as shown in **Figure 6b**), we integrate the compound content measurement results for all organs, roots, and leaves of 50 cultivated accessions of *S. miltiorrhiza* and provide dynamic charts to visualize the expression levels of major metabolites. For example, from the metabolite content measurement results for all organs, we can see that salvianolic acid B is the most expressed and accumulated metabolite across all *S. miltiorrhiza* lines, followed by rosmarinic acid, which also shows relatively high expression levels.

### 3.5 Protein module

**Figure 7:**
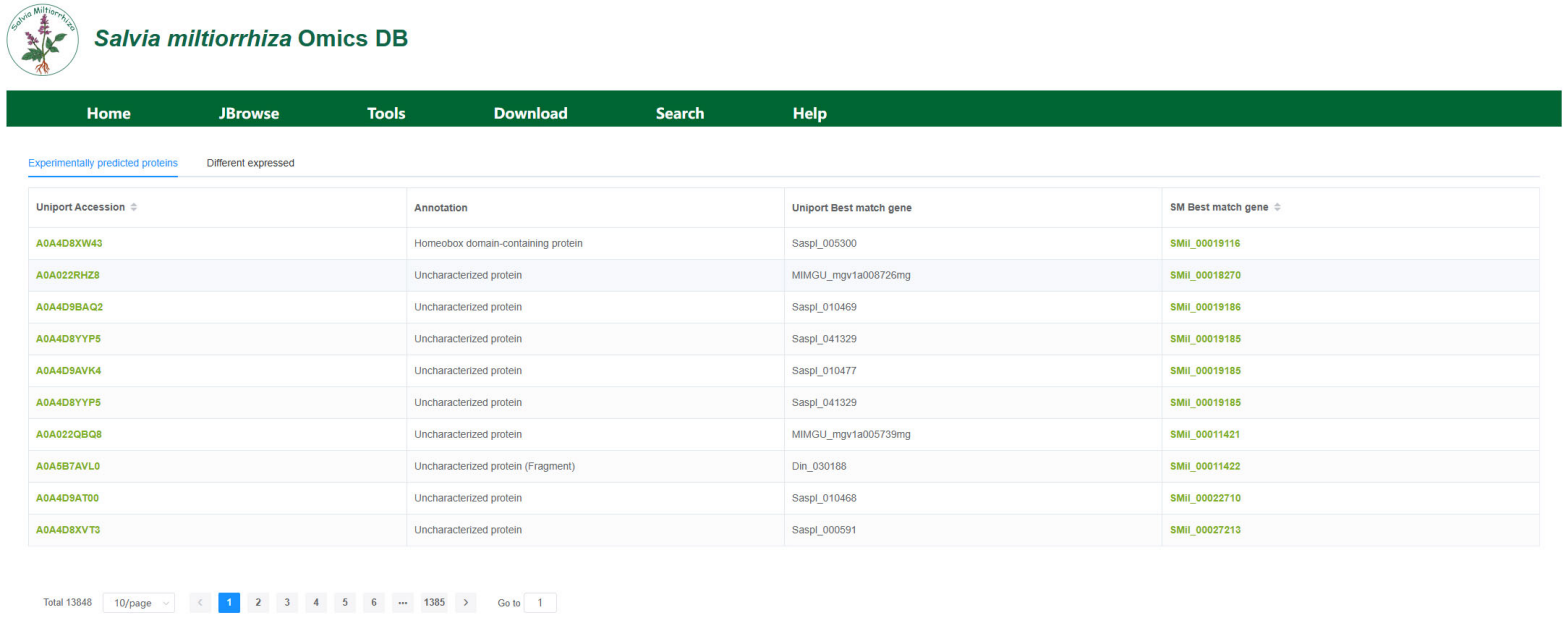
Protein Information Module. This section shows the gene IDs and names of the best-matching genes in the UniProt database and the current genome.

The protein module consists of the protein information and protein visualization sections. The protein information section, as shown in **Figure 7**. presents experimental predictions of proteins and validated protein expression data. We utilized 13,848 protein entries annotated in the UniProt database and compiled the corresponding gene IDs and names, both from the best matches in the UniProt database and the most matching genes in the current genome, along with the associated annotation information.

**Figure 8:**
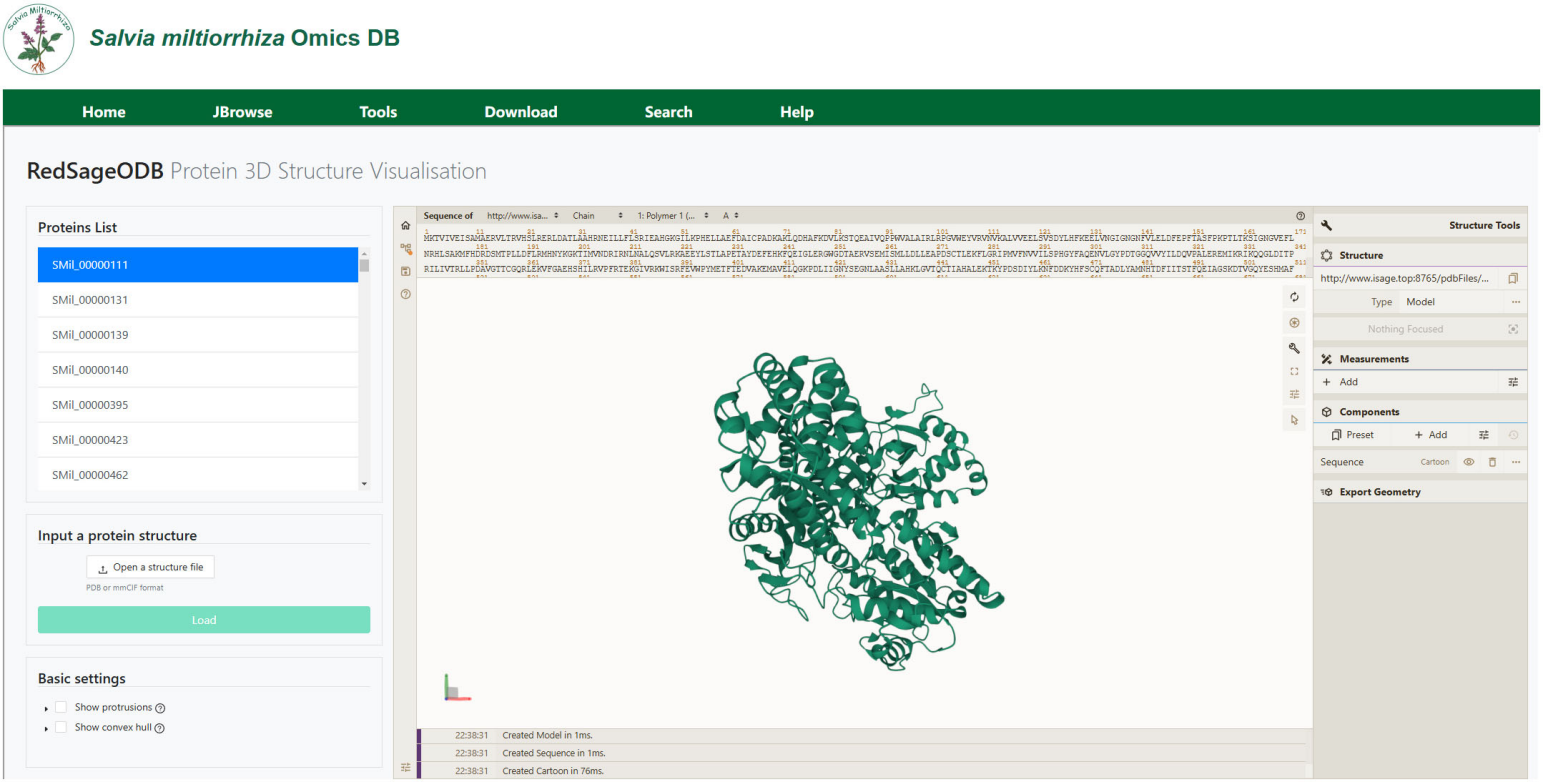
Example of predicted 3D structures of proteins expressed in *S. miltiorrhiza*.

In the protein visualization module, we implemented a customized Mol* viewer (Molstar) to display three-dimensional structures of 2,967 *S. miltiorrhiza* expression proteins predicted using the RoseTTAFold algorithm, as shown in **Figure 8**. Users can rotate or zoom in on the protein structures from various angles, and examine the position of each amino acid residues corresponding to its 3D structure within the entire protein. Additionally, users can upload their own protein structure files to view the visualization results and perform further analysis.

### 3.6 SmilODB tools

**Figure 9:**
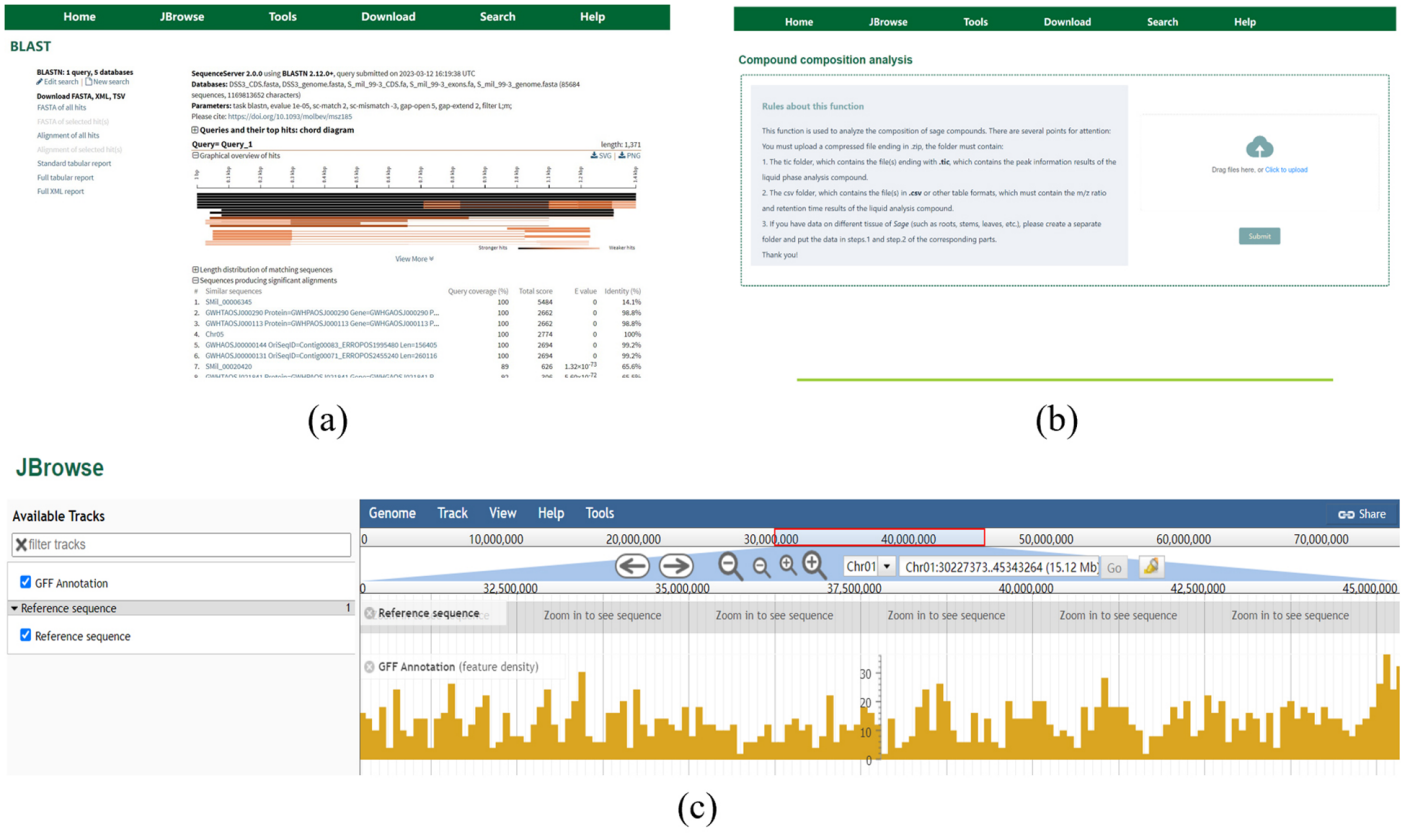
SmilODB Tools interface **(a)** The “BLAST” tool. **(b)** Compound analysis tool. **(c)** “Genome Browser” tool.

SmilODB integrates a suite of widely used bioinformatics tools into its navigation bar, offering comprehensive analytical and visualization functionalities that substantially enhance both database utility and user experience. First, as illustrated in **Figure 9a**, SmilODB incorporates a BLAST search tool that allows users to input or upload query sequences in FASTA format. By selecting suitable algorithms (e.g., BLASTN, BLASTP) and customizing parameters, users can perform sequence alignments against various internal and external databases. The tool provides detailed output, including similarity scores, E-values, and alignment statistics, offering valuable support for homologous sequence identification and comparative genomics. Second, SmilODB integrates the interactive JBrowse genome browser (**Figure 9b**), which enables users to navigate genomic data across different annotation tracks. The browser supports zooming, region dragging, and coordinate-based searches. Users can click on gene models to retrieve detailed information such as gene length, genomic coordinates, nucleotide and protein sequences, and functional annotations. This tool facilitates intuitive exploration of the *S. miltiorrhiza* genome and supports interactive visualization for gene-centric analysis. Third, SmilODB offers a compound analysis tool (**Figure 9c**) designed to support the upload and analysis of mass spectrometry (MS) data. Users can upload raw MS files for automated comparison against an integrated compound reference database. The system returns detailed analysis reports, including mass-to-charge ratio (m/z), retention time, and match scores, enabling efficient compound identification and facilitating metabolomic and functional studies. In addition to these tools, SmilODB provides direct access to downloadable genomic datasets via the Download section in the navigation bar. To assist users—especially first-time visitors—a Tutorial section is available, offering step-by-step instructions for each function of the platform. These integrated features collectively establish SmilODB as a multidimensional support platform for *S. miltiorrhiza* research.

## 4 Discussion

This study developed a comprehensive multi-omics database, SmilODB, specifically designed for the integration and analysis of *S. miltiorrhiza* genomic data. SmilODB not only overcomes the limitations of the existing SmGDB database (Zhou et al., 2022) in terms of data fragmentation and functionality, but also fills the gap for a comprehensive database dedicated to the study of the *S. miltiorrhiza* genome. By integrating genomic, transcriptomic, proteomic, and metabolomic data, SmilODB provides a user-friendly platform that supports multidimensional data querying, dynamic visualization, and functional annotation. This database significantly enhances the efficiency of researching genomic information related to *S. miltiorrhiza*, especially in analyzing complex biological processes and pharmacological mechanisms.

Compared to existing *S. miltiorrhiza* genomic databases (Zhou et al., 2022), SmilODB demonstrates three distinct advantages. First, it systematically integrates expanded multi-omics datasets [genomic, transcriptomic, and metabolomic data (Xu et al., 2016; Song et al., 2020)], enabling researchers to access comprehensive biological insights through a unified portal. Second, it provides transcriptome analysis and KEGG-annotated metabolic pathway diagrams for *S. miltiorrhiza* tissues, facilitating the exploration of pharmacological mechanisms by correlating gene expression with metabolite accumulation. Third, it introduces artificial intelligence to predict the three-dimensional structures of key proteins, thereby offering new technical support for deciphering essential biological processes in *S. miltiorrhiza* (Chaudhury et al., 2010; Veličković et al., 2017; Fuchs et al., 2020).

Despite these strengths, SmilODB still has areas for improvement. For instance, as the volume of data and research demands continue to grow, future updates will need to incorporate more machine learning-based automated analysis tools to address the expanding data processing needs (Sprang et al., 2022; Etcheverry et al., 2025).Concurrently, we plan to expand data retrieval modules to support downloadable transcriptomic and metabolomic datasets. This will assist researchers in uncovering the biosynthetic pathways and regulatory mechanisms of active components in *S. miltiorrhiza*, offering valuable insights for drug development.

SmilODB will continue to be updated and expanded, further integrating new multi-omics datasets (Pan et al., 2023),such as epigenomic data; developing more powerful metabolic network modeling tools; and expanding the 3D protein structure prediction module, in order to provide greater support for the applied research of *S. miltiorrhiza*.

## 5 Conclusions

In conclusion, *S. miltiorrhiza* is a valuable medicinal plant with immense pharmacological potential, yet the absence of a comprehensive and integrated multi-omics database has hindered the efficient analysis of its complex biological data. To address this gap, we developed SmilODB, a robust platform that consolidates genomics, transcriptomics, proteomics, and metabolomics data, offering a user-friendly interface for researchers to easily access, analyze, and visualize data. This database significantly enhances the study of *S. miltiorrhiza* by providing tools to explore its gene functions, protein structures, and metabolic pathways, promoting a deeper understanding of the plant’s biological processes and pharmacological mechanisms. Furthermore, SmilODB will be continually updated to incorporate new data, enhance protein structure predictions, and introduce additional features to support ongoing research. The development of such a comprehensive resource will facilitate the translation of *S. miltiorrhiza* research into practical applications, advancing its potential in therapeutic and pharmaceutical applications.

## Data Availability

All the pertinent data are accessible through the SmilODB website (http://www.isage.top:56789/#/home, accessed on 25 February 2025).
